# Supplementary material for: High-affinity peptides developed against calprotectin and their application as synthetic ligands in diagnostic assays
Source: Nat Commun. 2023 May 17;14:2774. doi: 10.1038/s41467-023-38075-7 (PMC10192418; doi:10.1038/s41467-023-38075-7)
Supplement: Supplementary file 1 — Supplementary Information [file 41467_2023_38075_MOESM1_ESM.pdf]

Supplementary Figures

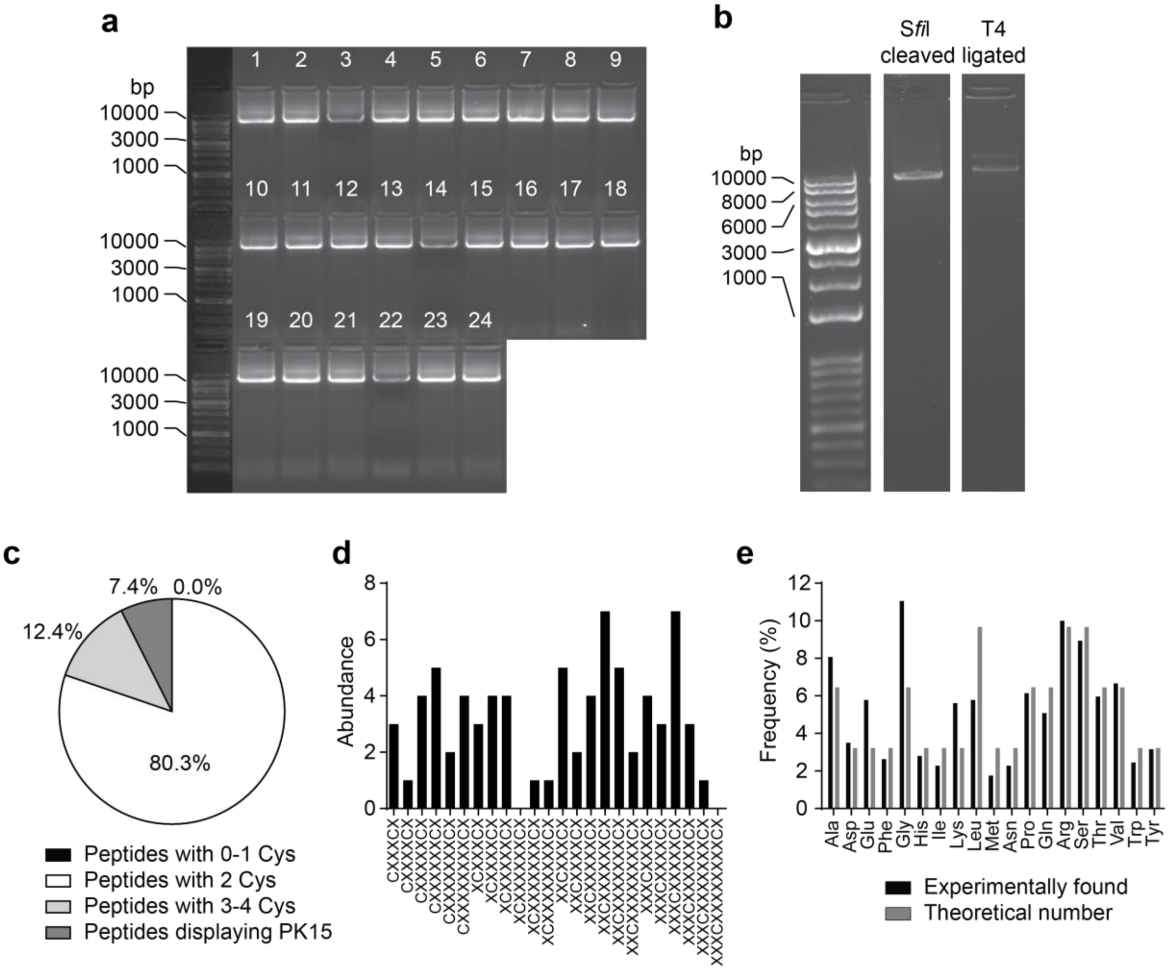

Supplementary Fig. 1. Cloning of phage display Library 3 by whole-plasmid PCR. a, PCR products obtained with 24 different degenerated primers and analyzed by agarose electrophoresis. Primer numbers are indicated. The contrast of the marker lane enhanced to make the faint bands visible. **b**, The 24 PCR products were pooled after purification, digested with S*fi*I, purified and analyzed before and after ligation with T4 ligase. The three lanes (cropped) were run on the same gel. **c**, Number of cysteines in 96 clones sequenced to evaluate the library quality. **d**, Abundance of the peptide formats in the 96 clones sequenced. **e**, Experimental and theoretical frequency of all amino acids (except cysteine) in the sequenced clones.

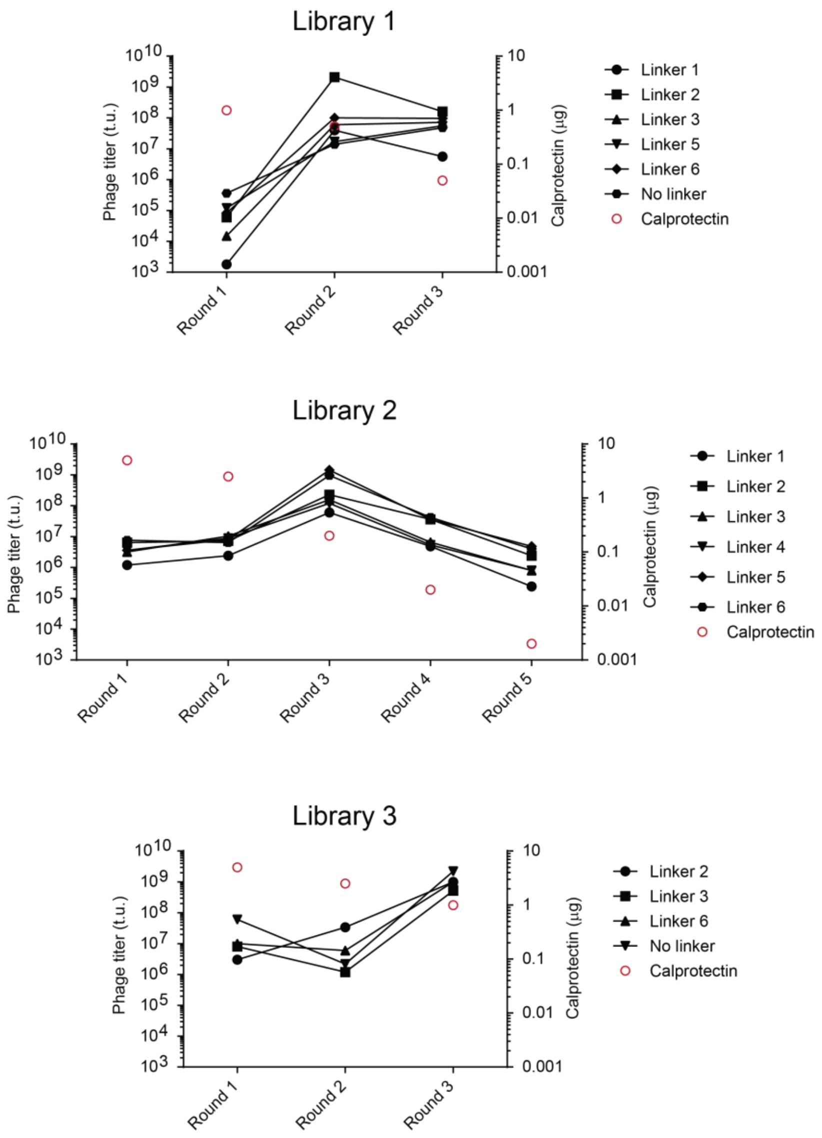

Supplementary Fig. 2. Phage display selections. Number of infective phage (t.u.) isolated in the various rounds of phage display selection. The quantity of biotinylated calprotectin tetramer used in the selection rounds was reduced over increasing rounds and is indicated in red.

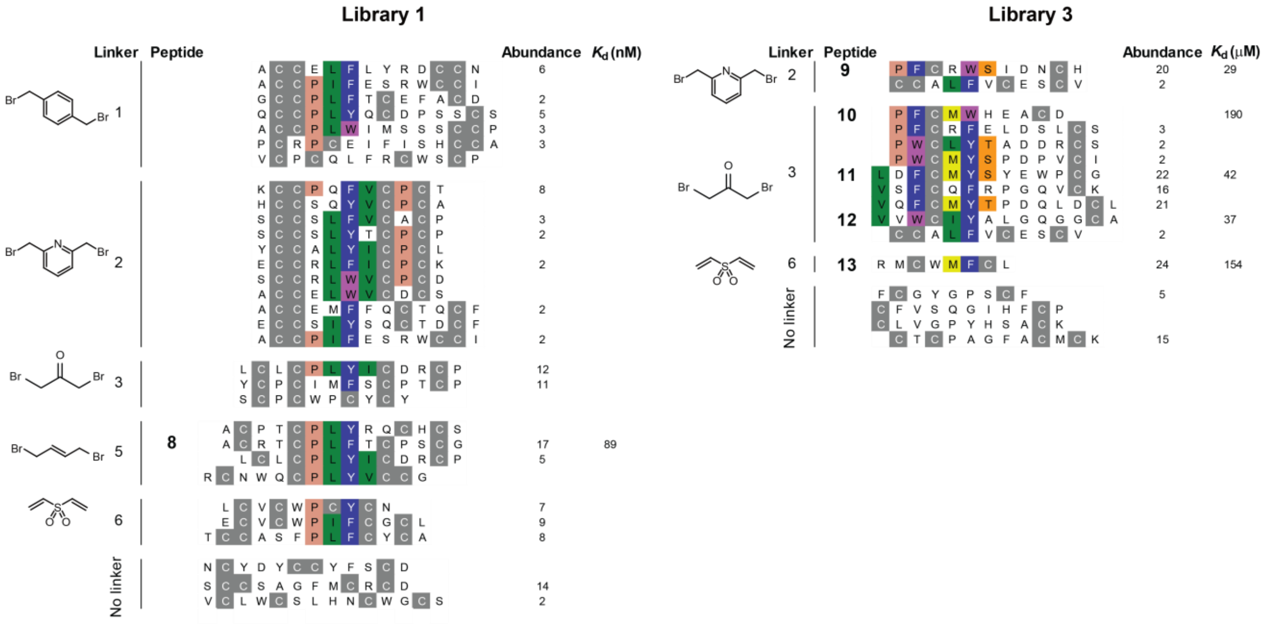

Supplementary Fig. 3. Phage selections with Library 1 and 3. Sequences isolated after three rounds of selection are shown. Amino acid similarities are highlighted in color, and the abundance of each peptide is indicated. Selected peptides were synthesized, cyclized and their affinity for calprotectin measured by FP. The indicated K_ds are for the binding to calprotectin in presence of calcium and thus the tetrameric form. For calculating the dissociation constants, we assumed that each calprotectin tetramer can bind two peptides, and thus calculated the K_ds using the concentration of B-RCAL protein in the assay (and not tetramer concentration).

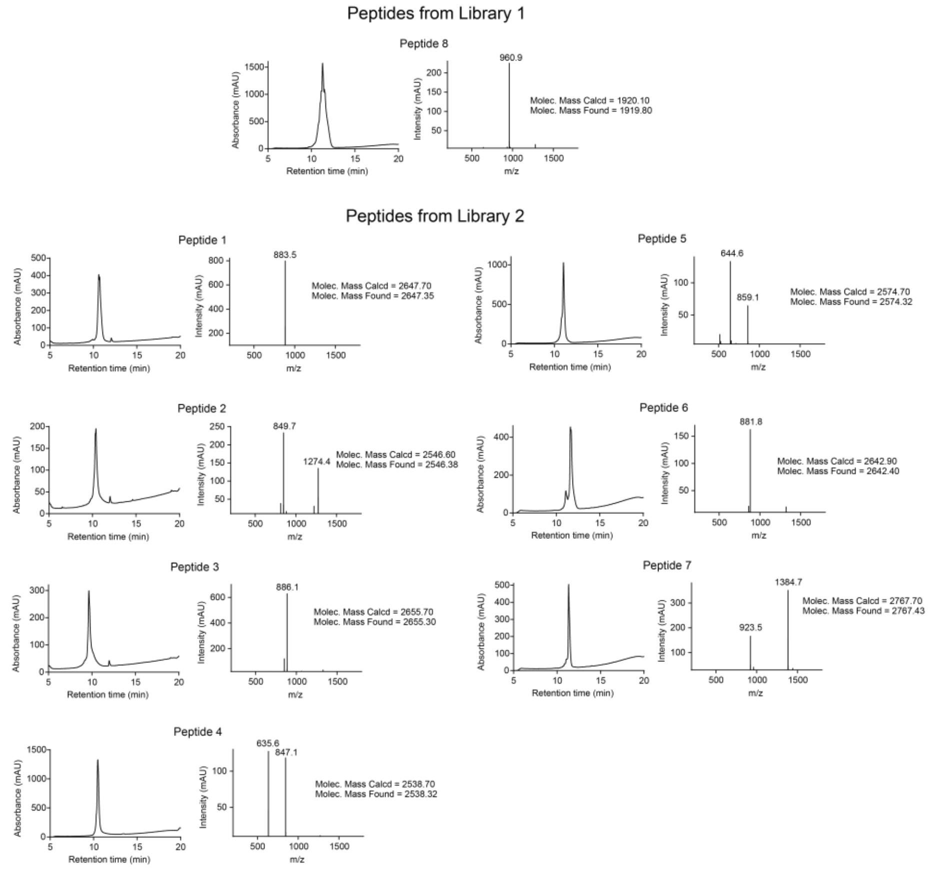

Supplementary Fig. 4. Purity and identity of peptides. Peptides were analyzed by analytical HPLC, where chromatograms are recorded at 220 nm in a 15-min linear gradient from 0 to 100% of MeCN (0.1% TFA) in H_2O/MeCN (95:5; 0.1% TFA). Masses were measured by ESI-MS. The expected and experimentally determined molecular masses are indicated. Peptides 1 to 8 were obtained by reacting unprotected peptides with the corresponding linker and HPLC-purification of the isomers. HPLC chromatograms and MS data are shown for the most active isomer of each peptide. The three isomers of Peptide 3 were synthesized by step-wise removal of the protecting groups and cyclization with the appropriate linker. The small peaks eluting around one minute before the desired peptides, observed for many of the Peptide 3 variants, were identified to be peptide with oxidized methionine.

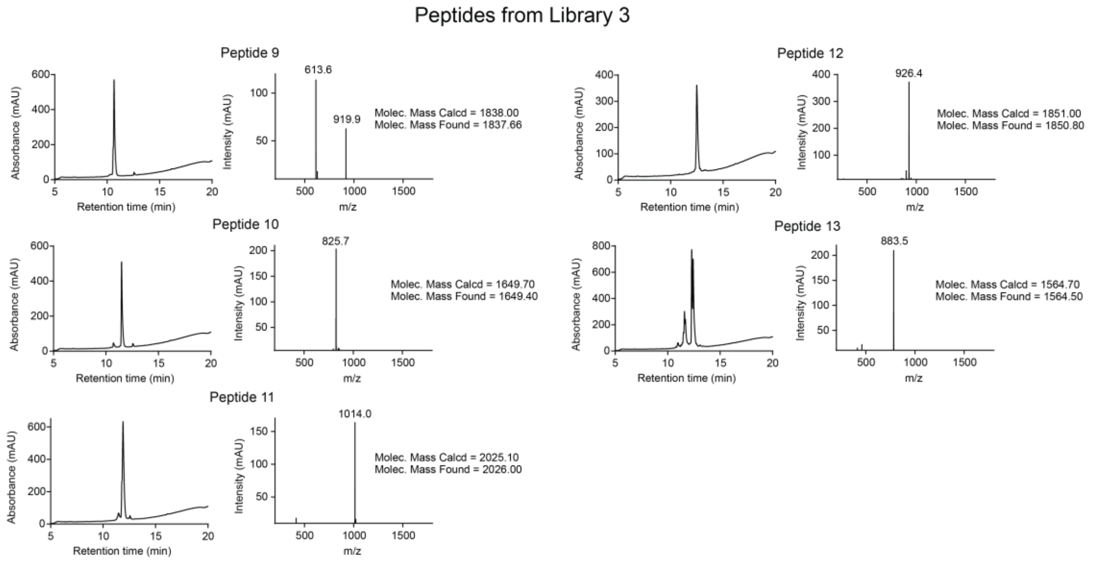

Supplementary Fig. 4. Continued.

Variants of Peptide 3

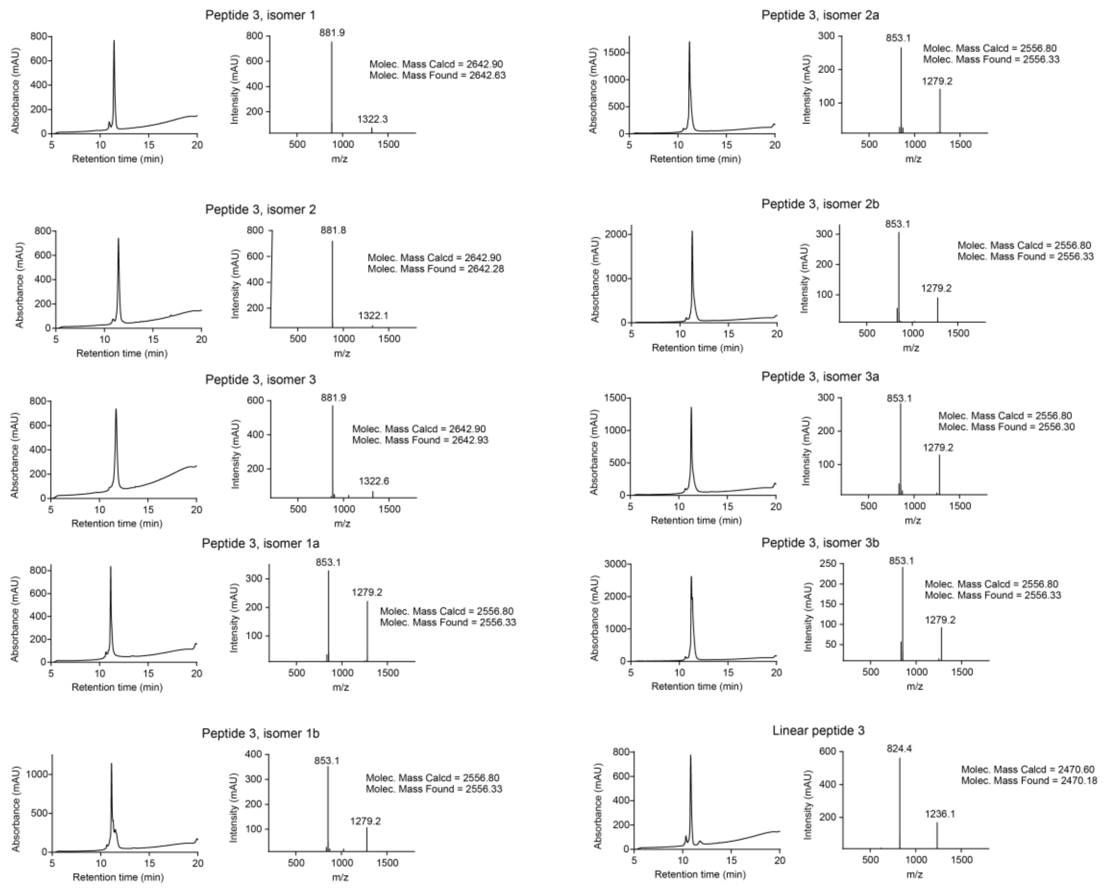

Supplementary Fig. 4. Continued.

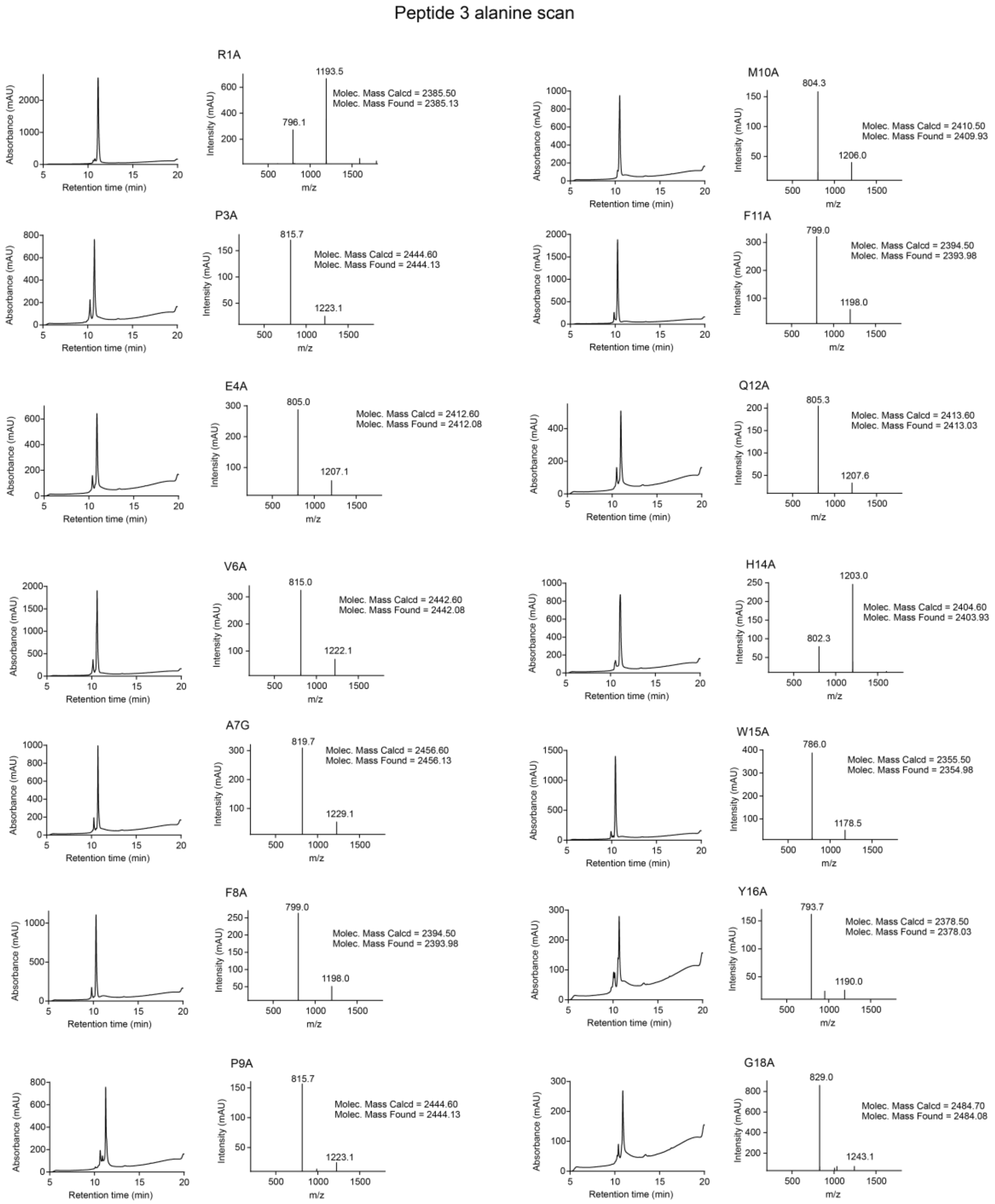

Supplementary Fig. 4. Continued.

Peptide 3 - biotin conjugates

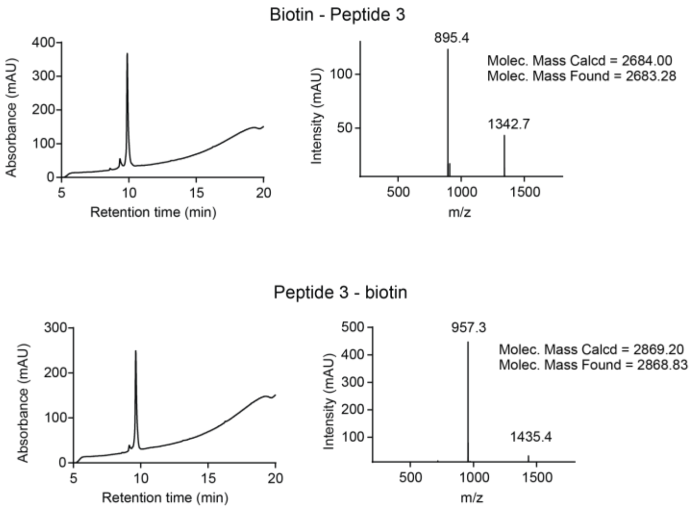

Supplementary Fig. 4. Continued.

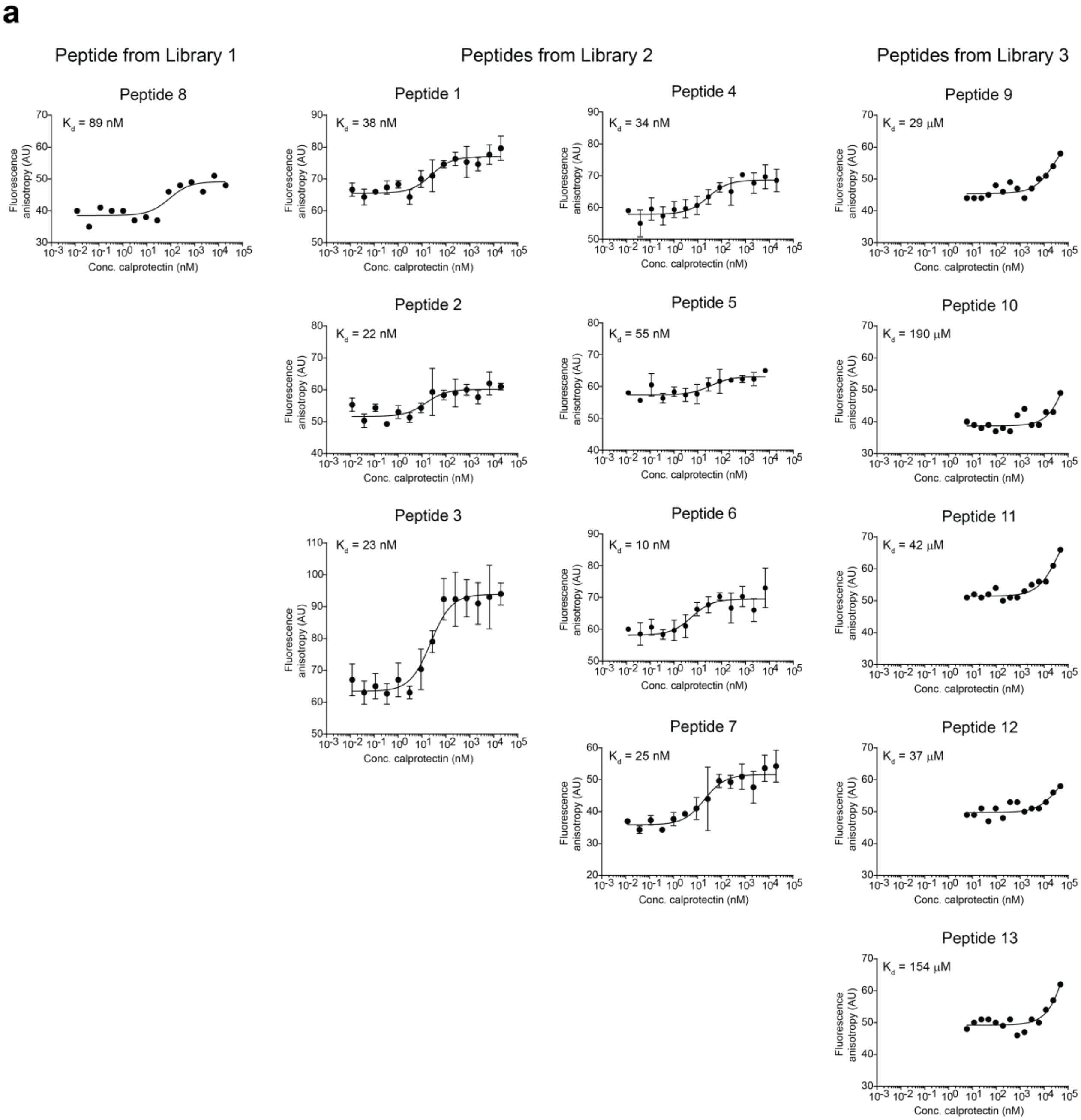

Supplementary Fig. 5. Affinity of peptides measured with FP assay. Fluorescein-labeled peptides (20 nM) were incubated with calprotectin in presence of calcium (2 mM) to induce tetramerization and the fluorescence anisotropy was measured. The calprotectin concentration is indicated as conc. of B-RCAL protein. For calculating the dissociation

constants, we assumed that each calprotectin tetramer can bind two peptides, and thus calculated the K_ds using the concentration of B-RCAL protein in the assay (and not the tetramer concentration). **a**, Peptides isolated from Libraries 1 to 3. For each peptide, the binding curve is shown for the isomers that showed the strongest binding. For peptides isolated from Library 2, three independent measurements were performed and mean values and SDs are indicated. For peptides from Libraries 1 and 3, that showed rather weak binding in the first measurement, the FP assay was not repeated. **b**, Linear Peptide 3 with amino acids exchanged individually to alanine. **c**, Linear Peptide 3 with amino acid Met10 mutated to leucine.

b Alanine scan of Peptide 3

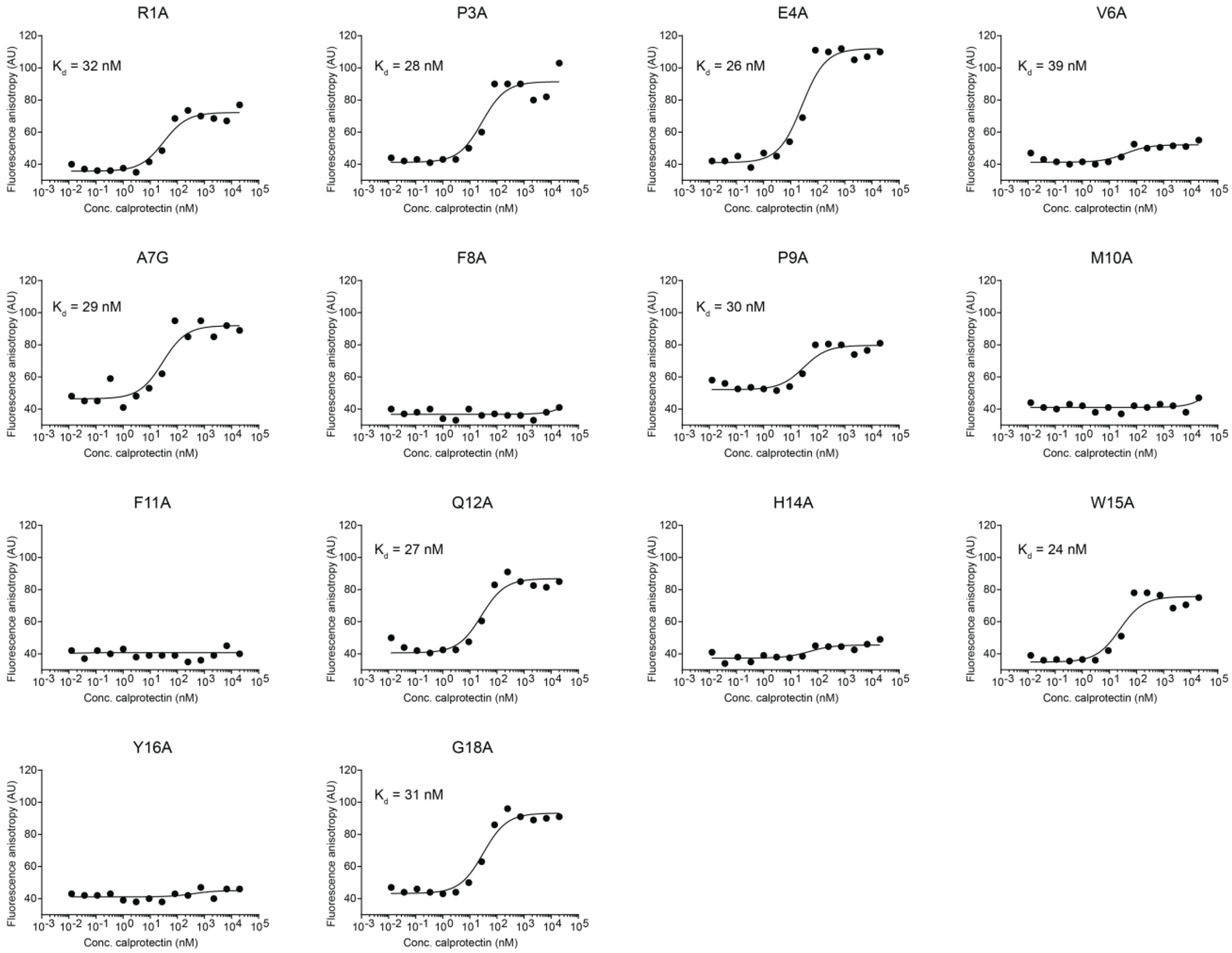

c Peptide 3 Met10Leu

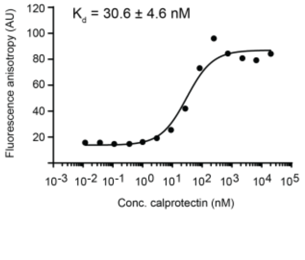

Supplementary Fig. 5. Continued.

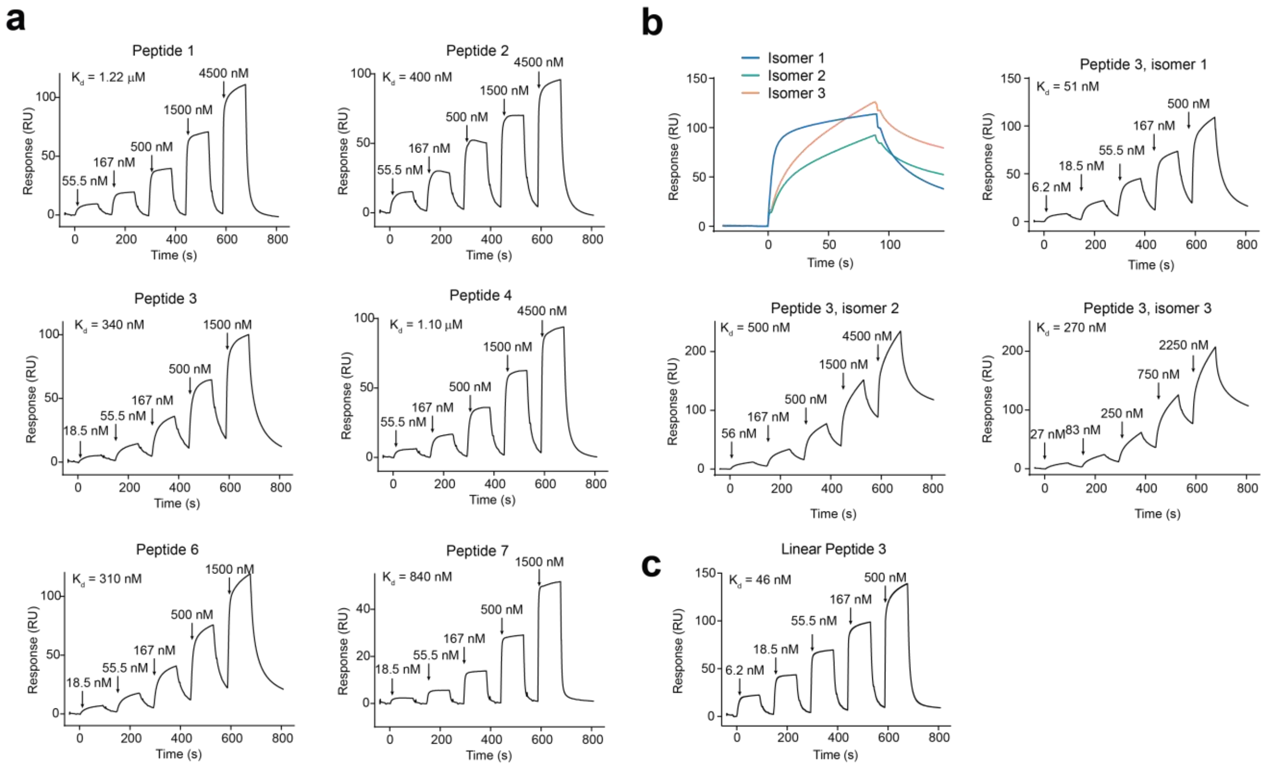

Supplementary Fig. 6. SPR sensorgrams of peptides binding to calprotectin tetramer. a, Single-cycle kinetic SPR sensorgrams of peptides from Library 2. **b**, Binding response and single-cycle kinetic SPR sensorgram of the three isomers of Peptide 3. **c**, Single-cycle kinetic SPR sensorgram of linear Peptide 3. Calprotectin was immobilized on the surface of the chip and the peptides were injected at the indicated concentrations. The K_d was calculated using a 1:1 fitting.

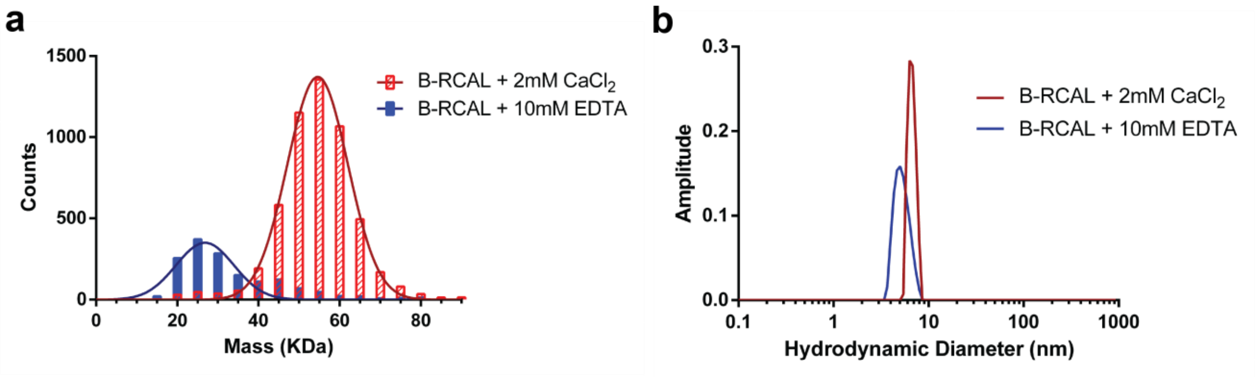

Supplementary Fig. 7. Analysis of the oligomeric state distribution of calprotectin in presence and absence of calcium ions. B-RCAL in buffer containing $CaCl_2$ or EDTA was analyzed by mass photometry (MP) (**a**) and dynamic light scattering (DLS) (**b**). EDTA was used to capture Ca^{2+} already present in the buffer of stock protein. The expected molecular weight and radius of B-RCAL dimer are 26.9 Da and 5.71 nm, respectively. The expected molecular weight and radius of B-RCAL tetramer are 53.8 Da and 6.82 nm, respectively. The molecular weights and hydrodynamic radii found by DLS and MP indicate that calprotectin forms a tetramer in presence of $CaCl_2$ and a dimer in absence (EDTA).

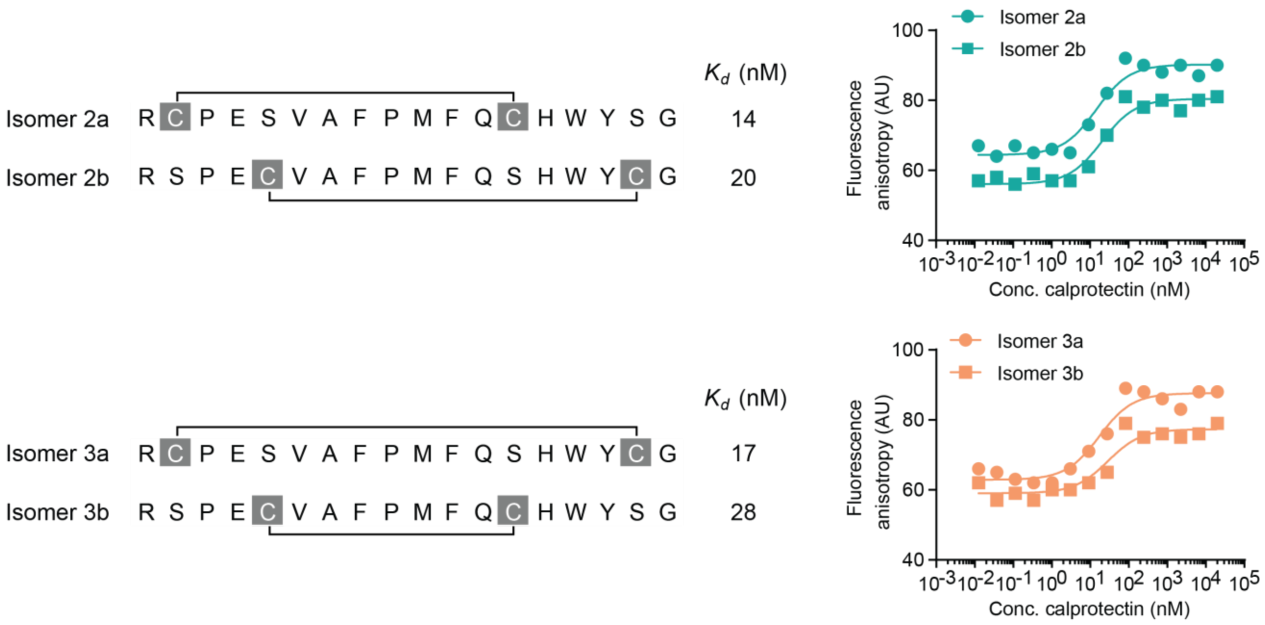

Supplementary Fig. 8. Singly-bridged peptides. Fluorescein-labelled peptides (20 nM) were incubated with calprotectin tetramer (2 mM calcium) and fluorescence anisotropy was measured. The calprotectin concentration is indicated as conc. of B-RCAL protein. For calculating the dissociation constants, we assumed that each calprotectin tetramer can bind two peptides, and thus calculated the K_ds using the concentration of B-RCAL protein in the assay (and not the tetramer concentration).

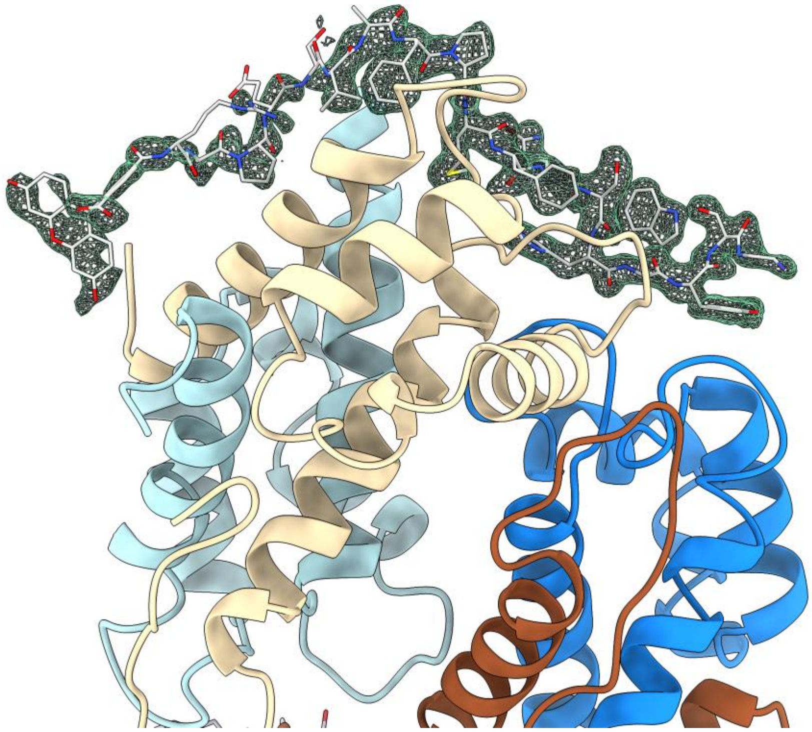

Supplementary Fig. 9. OMIT map. Running a 2Fo-Fc omit map contoured at 2.5 RMSD unambiguously proved that the additional electron density came from the peptide. The electron density of the Peptide 3 is shown as grid.

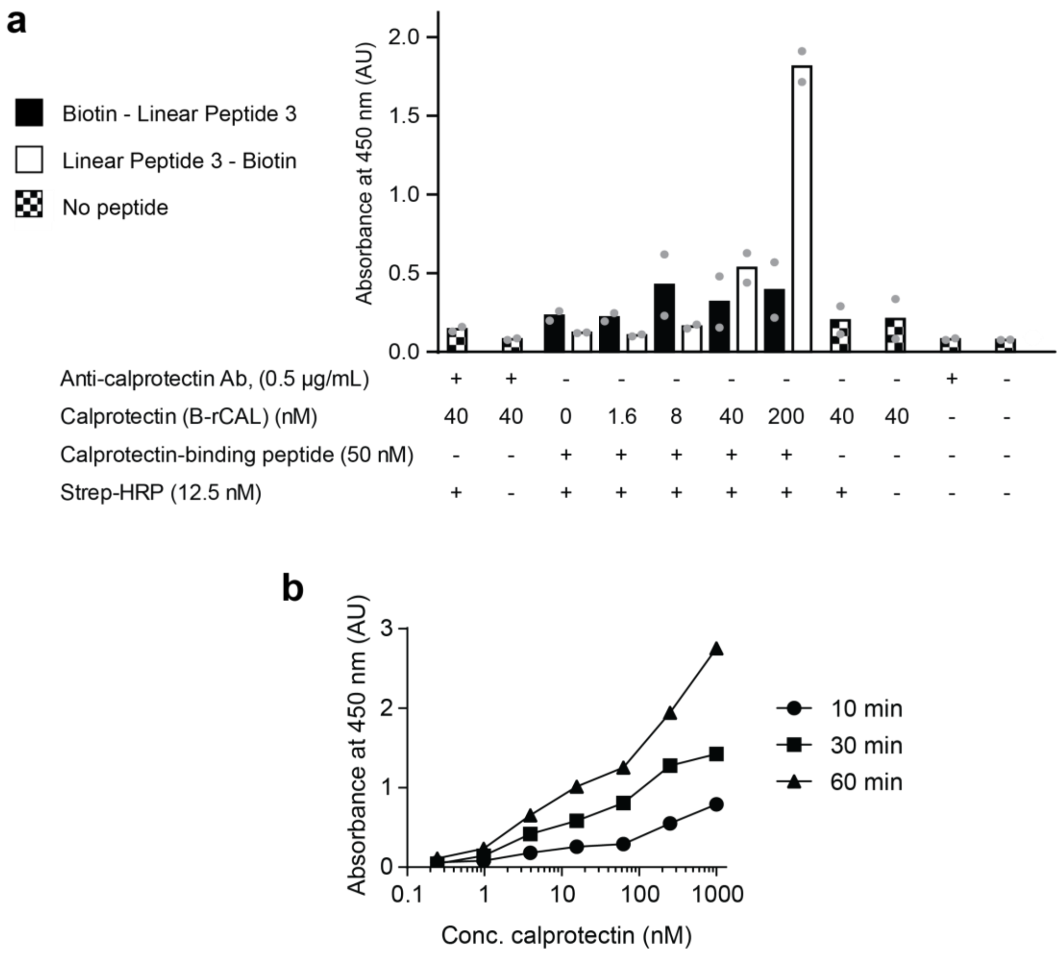

Supplementary Fig. 10. Detection of calprotectin by ELISA. a, Controls for the ELISA assay. Different concentrations of calprotectin were captured on immobilized anti-calprotectin antibody and detected with either of the two biotinylated peptides/Strep-HRP. Average values (bars) and individual values (dots) of two measurements are shown. **b**, Absorbance dependence on the incubation time of peptide with calprotectin. Graph shows three independent experiments where the only parameter modified is the incubation time between the Strep-HRP/peptide pre-mixture with the immobilized calprotectin. All the experiments were performed in duplicate and the mean values are indicated.

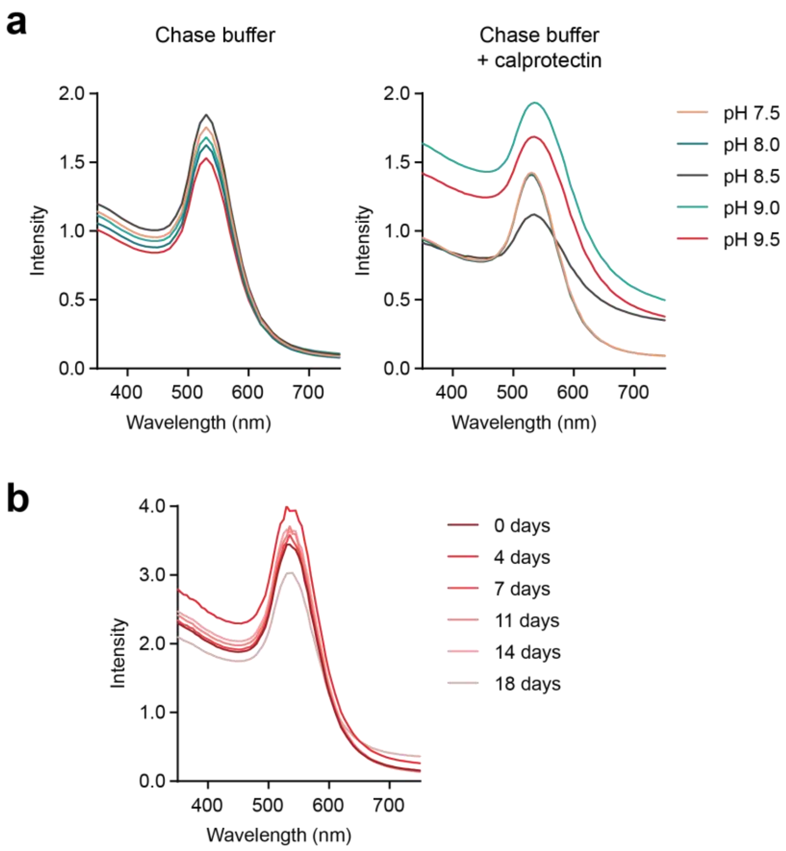

Supplementary Fig. 11. Stability of AuNP conjugated with linear Peptide 3-biotin. a, Stability at several pHs in chase buffer only or in the presence of calprotectin (5 μg/mL). **b**, Stability over time in 10 mM Tris, pH 8.0, with 10% sucrose and 1% Tween-20. Conjugated AuNP were kept at 4°C and the stability was assessed by measuring the UV-Vis profile at the indicated times.

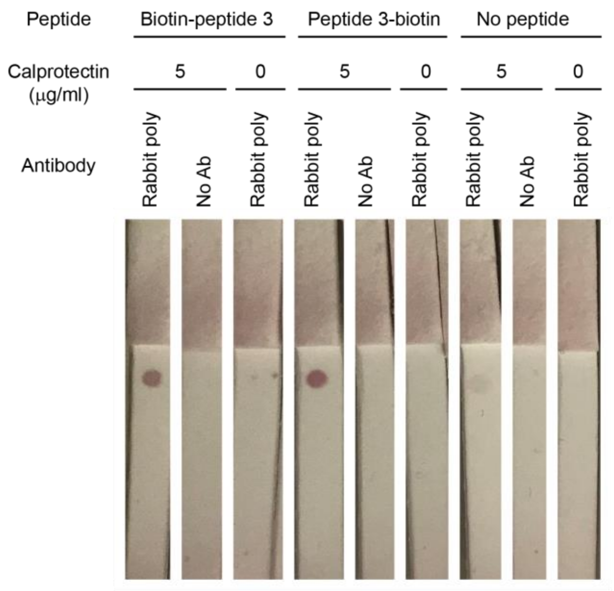

Supplementary Fig. 12. Dipstick LFA proof-of-concept. Photographs of the LF strips after 15 min incubation with the indicated conditions. Test dot was coated with/without the rabbit polyclonal antibody anti-calprotectin. Calprotectin was added at 0 or 5 μg/mL. Peptide 3, biotinylated at either end, was bound to the Strep-AuNPs.

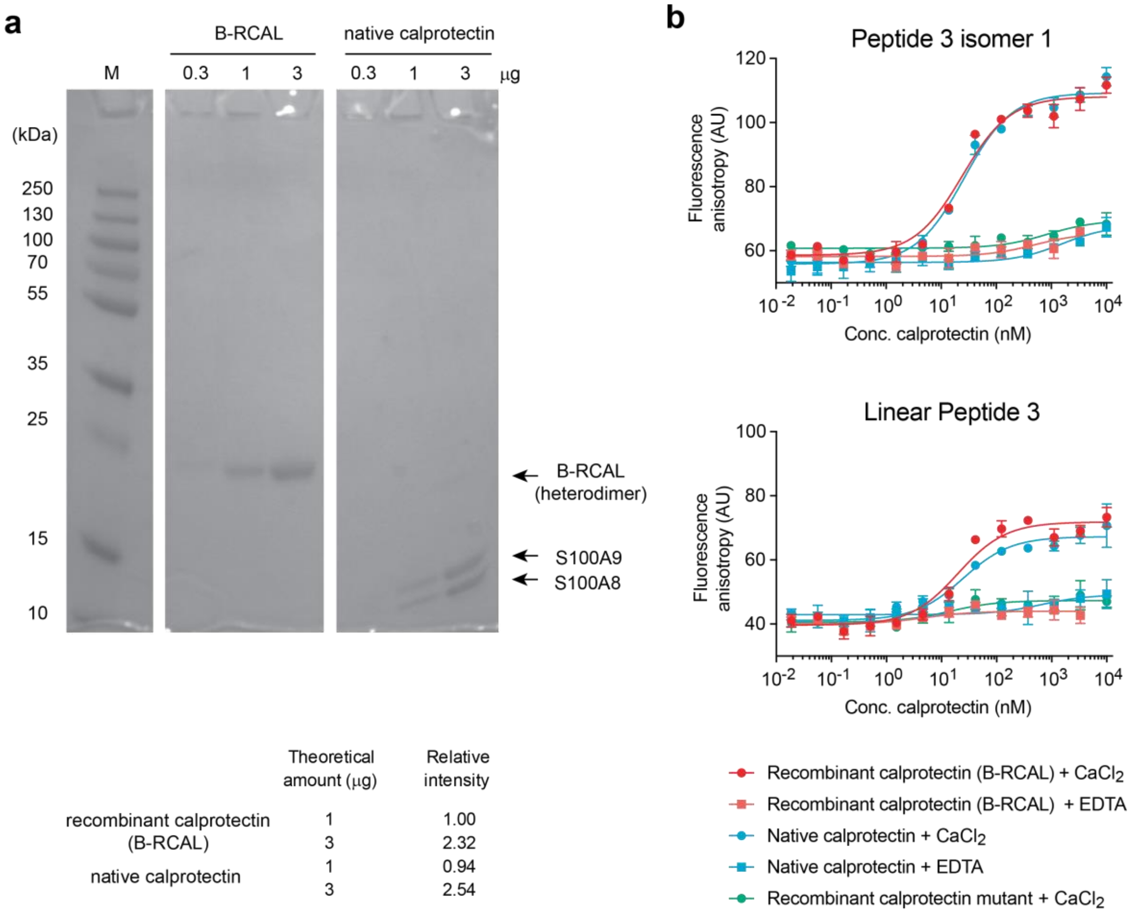

Supplementary Fig. 13. Binding of peptide to native calprotectin. Fluorescein-labeled peptides (20 nM) were incubated with different calprotectin species and the fluorescence anisotropy was measured. Native calprotectin is derived from granulocytes. Recombinant calprotectin mutant E78A is based on B-RCAL. It does not oligomerize into the tetrameric state in presence of calcium. FP buffer contained $CaCl_2$ (0.1 mM) or EDTA (1 mM). Mean values and SDs of three independent measurements are shown. For Peptide 3 isomer 1, the K_d values are 24 ± 3 nM for B-RCAL + $CaCl_2$, and 26 ± 2 nM for native calprotectin + Ca. For linear Peptide 3, the K_d values are 20 ± 4 nM for B-RCAL + $CaCl_2$, and 26 ± 13 nM for native calprotectin + $CaCl_2$.

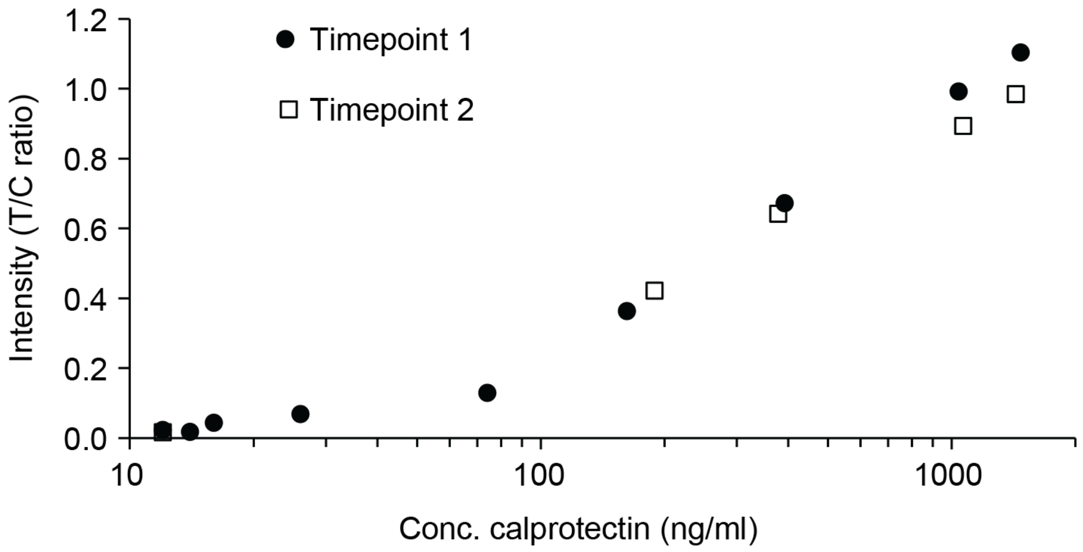

Supplementary Fig. 14. Stability of Peptide 3-AuNP-based LFA. The calprotectin concentration in serum spiked with different amounts of calprotectin was measured using Peptide 3-AuNP LFA cassettes immediately after their production and after 11 months of storage. The data of the time point 1 is essentially identical to the data shown in Figure 5e.

Supplementary Tables

Supplementary Table 1. DNA primers used for cloning Library 3 (5'→3').

No	Primer sequence
1	TGCGGCCCAGCCGGCCATGGCGTGTNNKNNKNNKTGTNNKGGTTCTGGCGCTGAAACTGTTG
2	TGCGGCCCAGCCGGCCATGGCGTGTNNKNNKNNKNNKTGTNNKGGTTCTGGCGCTGAAACTGTTG
3	TGCGGCCCAGCCGGCCATGGCGTGTNNKNNKNNKNNKNNKTGTNNKGGTTCTGGCGCTGAAACTGTTG
4	TGCGGCCCAGCCGGCCATGGCGTGTNNKNNKNNKNNKNNKNNKTGTNNKGGTTCTGGCGCTGAAACTGTTG
5	TGCGGCCCAGCCGGCCATGGCGTGTNNKNNKNNKNNKNNKNNKNNKTGTNNKGGTTCTGGCGCTGAAACTGTTG
6	TGCGGCCCAGCCGGCCATGGCGTGTNNKNNKNNKNNKNNKNNKNNKNNKTGTNNKGGTTCTGGCGCTGAAACTGTTG
7	TGCGGCCCAGCCGGCCATGGCGNNKTGTNNKNNKNNKTGTNNKGGTTCTGGCGCTGAAACTGTTG
8	TGCGGCCCAGCCGGCCATGGCGNNKTGTNNKNNKNNKNNKTGTNNKGGTTCTGGCGCTGAAACTGTTG
9	TGCGGCCCAGCCGGCCATGGCGNNKTGTNNKNNKNNKNNKNNKTGTNNKGGTTCTGGCGCTGAAACTGTTG
10	TGCGGCCCAGCCGGCCATGGCGNNKTGTNNKNNKNNKNNKNNKNNKTGTNNKGGTTCTGGCGCTGAAACTGTTG
11	TGCGGCCCAGCCGGCCATGGCGNNKTGTNNKNNKNNKNNKNNKNNKNNKTGTNNKGGTTCTGGCGCTGAAACTGTTG
12	TGCGGCCCAGCCGGCCATGGCGNNKTGTNNKNNKNNKNNKNNKNNKNNKNNKTGTNNKGGTTCTGGCGCTGAAACTGTTG
13	TGCGGCCCAGCCGGCCATGGCGNNKNNKTGTNNKNNKNNKTGTNNKGGTTCTGGCGCTGAAACTGTTG
14	TGCGGCCCAGCCGGCCATGGCGNNKNNKTGTNNKNNKNNKNNKTGTNNKGGTTCTGGCGCTGAAACTGTTG
15	TGCGGCCCAGCCGGCCATGGCGNNKNNKTGTNNKNNKNNKNNKNNKTGTNNKGGTTCTGGCGCTGAAACTGTTG
16	TGCGGCCCAGCCGGCCATGGCGNNKNNKTGTNNKNNKNNKNNKNNKNNKTGTNNKGGTTCTGGCGCTGAAACTGTTG
17	TGCGGCCCAGCCGGCCATGGCGNNKNNKTGTNNKNNKNNKNNKNNKNNKNNKTGTNNKGGTTCTGGCGCTGAAACTGTTG
18	TGCGGCCCAGCCGGCCATGGCGNNKNNKTGTNNKNNKNNKNNKNNKNNKNNKNNKTGTNNKGGTTCTGGCGCTGAAACTGTTG
19	TGCGGCCCAGCCGGCCATGGCGNNKNNKNNKTGTNNKNNKNNKTGTNNKGGTTCTGGCGCTGAAACTGTTG
20	TGCGGCCCAGCCGGCCATGGCGNNKNNKNNKTGTNNKNNKNNKNNKTGTNNKGGTTCTGGCGCTGAAACTGTTG
21	TGCGGCCCAGCCGGCCATGGCGNNKNNKNNKTGTNNKNNKNNKNNKNNKTGTNNKGGTTCTGGCGCTGAAACTGTTG
22	TGCGGCCCAGCCGGCCATGGCGNNKNNKNNKTGTNNKNNKNNKNNKNNKNNKTGTNNKGGTTCTGGCGCTGAAACTGTTG
23	TGCGGCCCAGCCGGCCATGGCGNNKNNKNNKTGTNNKNNKNNKNNKNNKNNKNNKTGTNNKGGTTCTGGCGCTGAAACTGTTG
24	TGCGGCCCAGCCGGCCATGGCGNNKNNKNNKTGTNNKNNKNNKNNKNNKNNKNNKNNKTGTNNKGGTTCTGGCGCTGAAACTGTTG

Supplementary Table 2. Data collection and refinement statistics for calprotectin complexed with peptide 3 (PDB ID: 7QUV).

	Calprotectin in complex with Peptide 3*
**Data collection**	
Space group	P 32 2 1
Unit cell	
a, b, c (Å)	50.789, 50.789, 148.752
α, β, γ (º)	90, 90, 120
Completeness (%)	97.65 (91.86)
Mean I/sigma(I)	21.34 (3.01)
Rmeas	0.077 (0.806)
Redundancy	10.5 (10.4)
**Refinement**	
Resolution (Å)	42.18 - 1.85 (1.916 - 1.85)
Unique reflections	19786 (1933)
Reflections used in refinement	19347 (1794)
Reflections used for R-free	1940 (177)
R-work	0.2205
R-free	0.2754
Number of non-hydrogen atoms	1916
macromolecules	1771
ligands	54
water	91
Protein residues	214
RMS (bonds, Å)	0.009
RMS (angles, º)	1.02
Wilson B-factor	25.62
Average B-factor	37.6
macromolecules	37.4
ligands	42.5
solvent	38.7

*Statistics for the highest-resolution shell are shown in parentheses.

Supplementary Table 3. Inter- and intra-molecular interactions between Peptide 3 and calprotectin. Optimal hydrogen bonds (HB) and salt bridges (SB) were defined using the web server PROFUNC and ChimeraX. The numbering of the residues of Peptide 3 is shown in Figure 3c.

**Linear Peptide 3** atom / residue	**Calprotectin** atom / residue	**Distance (Å)**	**Interaction**
O / Arg 1	ND2 / Asn 19 (S100A9)	3.06	HB
OG / Ser 2	OE2 / Glu 15 (S100A9)	2.81	HB
N / Val 6	O / Ser 88 (S100A8)	2.99	HB
O / Phe 8	ND1 / His 89 (S100A8)	2.76	HB
N / Phe 11	OD2 / Asp 32 (S100A9)	2.77	HB
O / Ser 13	ND2 / Asn 71 (S100A9)	3.04	HB
NE2 / His 14	OE2 / Glu 79 (S100A9)	3.30	SB
O / Trp 15	ND2 / Asn 71 (S100A9)	3.29	HB

**Linear Peptide 3** atom / residue	**Linear Peptide 3** atom / residue	**Distance (Å)**	**Interaction**
N / Trp 15	O / Ser 13	2.87	HB
N (NH2) / Gly 18	OH / Tyr 16	3.22	HB
