# Supplementary material for: High-affinity peptides developed against calprotectin and their application as synthetic ligands in diagnostic assays
Source: Nat Commun. 2023 May 17;14:2774. doi: 10.1038/s41467-023-38075-7 (PMC10192418; doi:10.1038/s41467-023-38075-7)
Supplement: Supplementary file 3 — Description of Additional Supplementary Files [file 41467_2023_38075_MOESM3_ESM.pdf]

Description of Additional Supplementary Files

File Name: Supplementary Movie 1

Description: Lateral flow assay applying an 80 µL droplet blood without calprotectin (negative control).

File Name: Supplementary Movie 2

Description: Lateral flow assay applying an 80 µL droplet blood containing calprotectin (950 ng/mL).

File Name: Supplementary Movie 3

Description: Lateral flow assay applying an 80 µL droplet serum without calprotectin (negative control).

File Name: Supplementary Movie 4

Description: Lateral flow assay applying an 80 µL droplet serum containing calprotectin (950 ng/mL).
