# Supplementary material for: High-affinity peptides developed against calprotectin and their application as synthetic ligands in diagnostic assays
Source: Nat Commun. 2023 May 17;14:2774. doi: 10.1038/s41467-023-38075-7 (PMC10192418; doi:10.1038/s41467-023-38075-7)
Supplement: Supplementary file 8 — Reporting Summary [file 41467_2023_38075_MOESM8_ESM.pdf]

nature portfolio

Corresponding author(s): C. Heinis, C-B. Gerhold

Last updated by author(s): 24.03.2023

Reporting Summary

Nature Portfolio wishes to improve the reproducibility of the work that we publish. This form provides structure for consistency and transparency in reporting. For further information on Nature Portfolio policies, see our Editorial Policies and the Editorial Policy Checklist.

Statistics

For all statistical analyses, confirm that the following items are present in the figure legend, table legend, main text, or Methods section.

n/a	Confirmed	
☐	☒	The exact sample size (*n*) for each experimental group/condition, given as a discrete number and unit of measurement
☐	☒	A statement on whether measurements were taken from distinct samples or whether the same sample was measured repeatedly
☒	☐	The statistical test(s) used AND whether they are one- or two-sided<br>*Only common tests should be described solely by name; describe more complex techniques in the Methods section.*
☒	☐	A description of all covariates tested
☒	☐	A description of any assumptions or corrections, such as tests of normality and adjustment for multiple comparisons
☐	☒	A full description of the statistical parameters including central tendency (e.g. means) or other basic estimates (e.g. regression coefficient) AND variation (e.g. standard deviation) or associated estimates of uncertainty (e.g. confidence intervals)
☒	☐	For null hypothesis testing, the test statistic (e.g. *F*, *t*, *r*) with confidence intervals, effect sizes, degrees of freedom and *P* value noted<br>*Give P values as exact values whenever suitable.*
☒	☐	For Bayesian analysis, information on the choice of priors and Markov chain Monte Carlo settings
☒	☐	For hierarchical and complex designs, identification of the appropriate level for tests and full reporting of outcomes
☒	☐	Estimates of effect sizes (e.g. Cohen's *d*, Pearson's *r*), indicating how they were calculated

Our web collection on statistics for biologists contains articles on many of the points above.

Software and code

Policy information about availability of computer code

Data collection	LC-MS Shimadzu 2020 instrument software, Biacore 8K Control Software, Tecan Infinite M200 Pro instrument software, Stunner instrument software, Refeyn AcquireMP (versions 2.3).
Data analysis	Excel (version 2016), Graphpad Prism (version 5), ChimeraX (version 1.5), XDS Program Package, Phenix (version 1.19.2), Coot (version 0.9.4.1), LC-MS Shimadzu 2020 instrument software (LabSolutions), Biacore 8K Evaluation Software, Tecan Infinite M200 Pro instrument software, Stunner instrument software, Refeyn DiscoverMP (version 2.3), Image J (version 1.54).

For manuscripts utilizing custom algorithms or software that are central to the research but not yet described in published literature, software must be made available to editors and reviewers. We strongly encourage code deposition in a community repository (e.g. GitHub). See the Nature Portfolio guidelines for submitting code & software for further information.

Data

Policy information about availability of data

All manuscripts must include a data availability statement. This statement should provide the following information, where applicable:

- Accession codes, unique identifiers, or web links for publicly available datasets
- A description of any restrictions on data availability
- For clinical datasets or third party data, please ensure that the statement adheres to our policy

Three supplementary tables and 14 supplementary figures are provided in the Supporting Information. Four movies showing the LFA assay are provided as Supporting Movies. Raw data are provided in a Source Data file. The atomic coordinates of linear Peptide 3 bound to calprotectin is deposited in the PDB (https://

www.rcsb.org) under the accession code 7QUV (http://doi.org/10.2210/pdb7QUV/pdb). The X-ray structure 4GGF (http://doi.org/10.2210/pdb4GGF/pdb) used in this work and published before can be found in the PDB too.

Human research participants

Policy information about studies involving human research participants and Sex and Gender in Research.

Reporting on sex and gender	9 healthy donors (2 male, 7 female), 18 rheumatoid arthritis patients (4 male, 14 female)
Population characteristics	Healthy donors: average age = 45, min = 20, max = 63<br>Rheumatoid arthritis patients: average age = 60, min = 31, max = 80
Recruitment	Serum patient samples were purchased by BÜHLMANN Laboratories from In.Vent Diagnostica GmbH (Henningsdorf, Germany) who had received ethical approval from the Freiburg Ethics Committee International (feki). Information on the recruitment procedure is now available.
Ethics oversight	The Freiburg Ethics Committee International (feki) has provided expert advice on the protocol and the patient information with declaration of consent according to ethical, legal and medical-scientific aspects. The Declaration of Helsinki as well as the international guidelines (e.g. the FDA Regulations, GEP and AWB guidelines) were taken into account.

Note that full information on the approval of the study protocol must also be provided in the manuscript.

Field-specific reporting

Please select the one below that is the best fit for your research. If you are not sure, read the appropriate sections before making your selection.

☒ Life sciences ☐ Behavioural & social sciences ☐ Ecological, evolutionary & environmental sciences

For a reference copy of the document with all sections, see nature.com/documents/nr-reporting-summary-flat.pdf

Life sciences study design

All studies must disclose on these points even when the disclosure is negative.

Sample size	Determination of calprotectin quantity in serum samples: 18 samples from 18 rheumatoid arthritis patients (one sample per patient); 9 samples from 9 healthy donors (one sample per patient). The sample size was chosen based on the maximal number of patient samples that was available. The sample size was not predetermined. A ROC curve analysis indicated a sufficiently large sample size.
Data exclusions	None
Replication	Peptides 1 to 7: mean values and SDs of three independent measurements.<br>Monocyclic and linear variants of Peptide 3: mean values and SDs of three independent measurements. In case of linear Peptide 3, the mean value and SD is based on five independent measurements.<br>ELISA with biotinylated Peptide 3: two independent measurements.<br>Calibration curve for recombinant calprotectin analyzed by LFA: mean values and SDs of five independent measurements.<br>Comparison of native and recombinant calprotectin in FP assay: mean values and SDs of three independent measurements.<br>Calibration curve for native calprotectin in serum analyzed by LFA: mean values and SDs of five independent measurements.<br>All attempts at replication were successful. All experiments not listed above were performed once.
Randomization	Randomization was not performed in this study as none of the experiments required comparison of two methods/procedures.
Blinding	Blinding was not applied in this study as all measurements were performed by instruments and the outcome could thus not be biased.

Reporting for specific materials, systems and methods

We require information from authors about some types of materials, experimental systems and methods used in many studies. Here, indicate whether each material, system or method listed is relevant to your study. If you are not sure if a list item applies to your research, read the appropriate section before selecting a response.

Materials & experimental systems

n/a	Involved in the study
☐	☒ Antibodies
☒	☐ Eukaryotic cell lines
☒	☐ Palaeontology and archaeology
☒	☐ Animals and other organisms
☒	☐ Clinical data
☒	☐ Dual use research of concern

Methods

n/a	Involved in the study
☒	☐ ChIP-seq
☒	☐ Flow cytometry
☒	☐ MRI-based neuroimaging

Antibodies

Antibodies used	Anti-calprotectin rabbit polyclonal antibody was purchased from BÜHLMANN Laboratories AG (type: 300135, lot number: 202104).
Validation	No validation statement was available from the provider for the polyclonal rabbit antibody (type: 300135, lot number: 202104) and it was thus validated experimentally in an ELISA experiment. The following controls were applied and they confirmed that the antibody was specifically binding to calprotectin: 1) no antibody, 2) no calprotecin, 3) no peptide (Supplementary Figure 10a).
